# Exosomes in Nephropathies: A Rich Source of Novel Biomarkers

**DOI:** 10.1155/2020/8897833

**Published:** 2020-08-12

**Authors:** Christos Masaoutis, Samer Al Besher, Ioannis Koutroulis, Stamatios Theocharis

**Affiliations:** ^1^First Department of Pathology, Medical School, National and Kapodistrian University of Athens, 75, Mikras Asias street, Bld 10, Goudi, 11527 Athens, Greece; ^2^Children's National Hospital, Division of Emergency Medicine and Center for Genetic Medicine, George Washington University School of Medicine and Health Sciences, 111 Michigan Ave. NW, Washington, DC 20010, USA

## Abstract

The biomarkers commonly utilized in diagnostic evaluations of kidney disease suffer from low sensitivity, especially in the early stages of renal damage. On the other hand, obtaining a renal biopsy to augment clinical decision making can lead to potentially serious complications. In order to overcome the shortcomings of currently available diagnostic tools, recent studies suggest that exosomes, cell-secreted extracellular vesicles containing a large array of active molecules to facilitate cell-to-cell communication, may represent a rich source of novel disease biomarkers. Because of their endocytic origin, exosomes carry markers typical for their parent cells, which could permit the localization of biochemical cellular alterations in specific kidney compartments. Different types of exosomes can be isolated from noninvasively obtained biofluids; however, in the context of kidney disease, evidence has emerged on the role of urinary exosomes in the diagnostic and predictive modeling of renal pathology. The current review summarizes the potential application of exosomes in the detection of acute and chronic inflammatory, metabolic, degenerative, and genetic renal diseases.

## 1. Introduction

Kidney disease, which encompasses various acute, chronic, or end-stage conditions, incurs a considerable health burden due to high prevalence and costly management [1]. While most cases of renal dysfunction are attributed to diabetes and hypertension, other inflammatory, immune-mediated, and genetic conditions have been implicated in kidney damage. A timely and accurate diagnosis is crucial for improved outcomes. Renal biopsy is an invaluable diagnostic tool for the establishment of the exact diagnosis and can aid in determining a prognosis and likelihood of response to treatment. As a result of the invasive technique used in obtaining the tissue samples, complications are numerous and can range from acute bleeding to the loss of the biopsied kidney. Renal biopsies are contraindicated in cases of increased bleeding risk, solitary kidneys, or renal anatomical abnormalities making diagnostic efforts in those cases very challenging [2].

The conventional biomarkers or renal disease in clinical practice are suboptimal: serum creatinine is limited by poor sensitivity in mild-to-moderate kidney failure and eGFR by its dependence on creatinine measurement in the early stage of renal dysfunction [3]; in addition, recent data question microalbuminuria as a reliable predictor of progression to end-stage renal disease [3].

Exosomes are bilipid membrane-bound vesicles measuring 40-120 nm in diameter; they are distinct from other extracellular vesicles (EVs) such as microvesicles and apoptotic bodies because their biogenesis is linked to the endosomal pathway. They are generated by an inward blebbing of the endosomal membrane that produces multivesicular bodies (MVBs), which are then fused with the plasma membrane and released via exocytosis (EL [4]). The exosomal cargo includes a variety of proteins, nucleic acids, lipids, and metabolites depending on their cell of origin and microenvironmental factors. All exosomes are highly enriched in proteins such as annexins, tetraspanins, and flotillin [5], which can be targeted in the process of exosome isolation and purification. The contents packaged into exosomes constitute intercellular mediators that can regulate certain physiologic processes including innate immunity, coagulation, spermatogenesis, central nervous system functions [6] , and bone remodeling [7]. In cancer biology, exosomes favor tumor progression by conditioning tumor microenvironment as well as remote premetastatic sites termed “premetastatic niches” [8] and can serve as liquid biopsies for various types of cancer [9, 10]. Recently, there has been an increasing interest in identifying exosomal biomarkers for nonneoplastic diseases [11–14].

There are multiple methods of exosome isolation. Centrifugation-based techniques, differential centrifugation, and density gradient ultracentrifugation are considered the gold standard. Differential centrifugation involves multiple steps of increasing centrifugation speed to first remove cells, apoptotic debris, and subsequently larger vesicles, so as to ultimately precipitate exosomes. Coprecipitation of EVs with apoptotic bodies and protein aggregates may occur. A way to avoid this is using a sucrose density gradient with centrifugation steps, separating the vesicles according to flotation density [15]. Filtration-based techniques, which separate vesicles depending on size and molecular weight, can be used independently, or as a replacement of the first two spins in differential centrifugation, so as to increase purity [15]. Tangential flow filtration is a technique that combines membrane filtration and flow, whereby the exosome-containing fluid flows tangentially across the membrane surface [16]. Size-exclusion chromatography is another size-based technique; it consists in EVs passing through diluted porous particles instead of a membrane, which results in different elution times for vesicles depending on whether they are small enough to enter the pores [15]. The best technique by far is the combination of tangential flow filtration and size-exclusion chromatography. Immunoaffinity-based separation takes advantage of exosomal membrane proteins, usually members of the tetraspanin family, such as CD9, CD63, and CD81 [15], and tissue-specific surface proteins when isolation of tissue-specific exosomes is desired, such as FABP4 for adipocyte-derived EVs using Western Blots [17]. Antibody-coated magnetic beads are commonly applied [18]. An elution buffer is required to release the exosomes from immunocomplexes [15]. Polymer-based precipitation methods consist in mixing the exosome-containing fluids with a polymer solution, usually polyethylene glycol, followed by recovering of the precipitated exosomes with low-speed centrifugation [15]. More recently, miniaturized microfluidic apparatuses using immunoaffinity-based or size-dependent separation techniques, or even contact-free particle sorting mechanisms (e.g., elastic lift force, acoustic, and dielectrophoresis), have been developed [15]. Nowadays, progress in the analytical procedures on exosome isolation bioassays proved helpful for better quantification of disease-specific exosomes in clinical samples [18].

The actual disease biomarkers are the miRNAs or proteins carried by the exosomes (exosomal cargo). Those miRNAs can be analyzed by RNA sequencing. There are currently bedside RNA sequencing techniques that give results within a few hours [19, 20].

Human models of renal disease have demonstrated that kidney damage is primarily driven by immune dysregulation and alterations in hemostasis, vascular integrity, and matrix modulation that are regulated by exosomes [21]. Circulating exosomes, capable of traversing basement membranes, are excreted in the urine and reuptaken by the collecting duct cells in a vasopressin-dependent manner. Renal tubular- and glomerular-derived exosomes are thought to participate in renal clearance and tissue regeneration [21]. Exosomes entrapped in the polymeric Tamm-Horsfall protein are hypothesized to mediate effects along tubular lumina, for instance, inducing the expression of proximal tubular proteins aquaporin-1 and glutaminase in downstream segments of the nephron [22].

The potential of exosomes to serve as therapeutic agents or drug delivery vehicles in chronic kidney disease [23] to alleviate systemic consequences [24, 25] makes them ideal treatment candidates. Multiple studies using preclinical, clinical, and ex vivo models have examined possible therapeutic applications of exosomes in diabetic nephropathy [26], hypertension-related cardiorenal syndrome [27], acute kidney injury [28, 29], IgA nephropathy [30], cadmium nephropathy [31], obstructive kidney disease [32], and ischemia/reperfusion injury [33].

MicroRNAs (miRNAs) are small noncoding RNAs that regulate gene expression mainly through RNA silencing [34]. Exosomes transport miRNA clusters which mediate autocrine and paracrine effects in target sites [34]. In chronic kidney disease, miRNAs are implicated in fibrosis, podocyte damage and apoptosis, mesangial cell hypertrophy or proliferation, and oxidative stress and inflammation [35]. Abundant miRNAs have been associated with kidney disease. Despite discrepancies in the literature, certain miRNAs seem to be consistently dysregulated, such as miR-21-5p, miR-29a-3p, miR-126-3p, miR-192-5p, miR-214-3p, and miR-342-3p in diabetic kidney disease [36]. Interestingly, amplification-free detection methods of exosomal miRNAs have been developed [37].

Exosomal cargo which determines cell-targeting can give us a wealth of information about the original cytosolic environment and relevant biochemical changes and also serves as a potential source of biomarkers. In this review, we aim to synthesize published data from human studies to date. The candidate biomarkers are presented in Tables 1–4 by potential clinical utility and are discussed in the text below by clinical condition.

## 2. Materials and Methods

We searched the online MEDLINE® database of the U.S. National Library of Medicine with the complex term (exosomes OR “extracellular vesicles”) AND (“kidney disease” OR “renal disease” OR “renal transplantation” OR “renal transplant” OR “renal failure” OR “kidney injury” OR nephritis OR “nephrotic syndrome” OR “nephritic syndrome”) which produced 393 results (last assessed on April 27th, 2020). Articles referring to “extracellular vesicles” were included as long as the described experimental method included exosomal isolation. Ultimately, the literature cited herein includes 95 peer-reviewed, original articles of studies in humans published in English. We used simple narrative analysis to summarize the data from the studies selected for review.

## 3. Results

### 3.1. Chronic Kidney Disease

Chronic kidney disease (CKD), which is characterized by the gradual irreversible deterioration of kidney function, is a multifactorial condition caused mainly by metabolic and inflammatory changes and is typically diagnosed and staged based on the estimated glomerular filtration rate (eGFR). In CKD, urinary exosomal cargo is characterized by higher levels of ceruloplasmin [38] and the overexpression of miR-181a-5p [39] and miR-451 [40] compared to healthy controls. Mir-181a-5p has been found to downregulate lipid metabolism regulator PPAR*α* [41] and is in silico predicted to downregulate MAT2A, TIMP3, and LGSF11 [42]; miR-451 downregulates YWHAZ and CAB39, which could be implicated in renal fibrosis and mesangial hypertrophy [40]. These biomarkers are identifiable early in CKD, and more specifically, ceruloplasmin can be detected at the premicroalbuminuric stage [38]. It is suggested that the extent of impairment of specific parts of the nephron is dependent on the underlying causative factors and disease stage. Studies have shown that podocyte injury may be related to a higher urinary concentration of exosomes expressing podocytal markers nephrin and podocalyxin [43], or containing higher amounts of miR-21 [44]. Renal fibrosis is the hallmark of permanent damage in CKD and has been associated with higher levels of miR-200b [45] and lower levels of miR-29c [46] as well as CD2AP mRNA [47].

### 3.2. Diabetic Nephropathy

Hyperglycemia secondary to diabetic nephropathy gradually damages all compartments of the kidney, beginning with glomerular capillary dysfunction with hyperfiltration and microalbuminuria and ultimately leading to interstitial fibrosis, tubular atrophy, and interstitial inflammation in advanced disease. Evidence of exosome-mediated podocytal injury is evident by either increased Elf3 protein [48] or WT1, podocin, Actn4, CD2AP, and nephrin mRNA [49]. Lower concentrations of mitochondria-specific metabolites such as 3-hydroxyisovalerate, citric acid, and 2-ethyl hydracrylic acid suggest mitochondrial dysfunction [50] that might be responsible for energy production dysregulation. Recognition of incipient damage is important due to the lack of early clinical manifestations. Other potential biomarkers for diabetic nephropathy include miR-21-5p [51], miR-15b, miR-34a, miR-636 [52], MASP2, CALB1 [53], myeloblastin, elafin, cystatin B, and neutrophil gelatinase-associated lipocalin [54], all of which increase in the presence of the condition. Decreased levels of miR-30b-5p [51], S100A8, S100A9 [53] and regucalcin [55] have also been described in diabetic nephropathy. The urinary exosomal miRNA profile [56], uromodulin mRNA levels [57], and C-megalin content [58] seem to be correlated with the degree of albuminuria.

### 3.3. Hypertensive Nephropathy

Hypertensive nephropathy is the result of either long-standing essential hypertension causing vascular-glomerular damage and remodeling or a primary renovascular lesion leading to renal hypoperfusion and secondary hypertension. The level of urinary exosomal plasmalemma vesicle-associated protein (PLVAP), a protein expressed in the peritubular capillaries, is associated with clinical measurements such as blood pressure and eGFR, and also the histological count of peritubular capillaries and degree of fibrosis in renal patients with essential or renovascular hypertension. This association makes PLVAP a potentially specific biomarker of microcirculation injury [59]. Urinary exosomes positive for nephrin and podocalyxin, proteins normally expressed in podocytes, have been isolated in the urine of patients with renovascular hypertension, indicating podocytal damage [60].

### 3.4. Acute Kidney Injury

Preliminary data from small clinical studies in critical care medicine have identified two urinary exosomal proteins as candidate biomarkers of acute kidney injury (AKI): (i) activating transcriptional factor 3 (ATF3), which is activated in models of ischemic reperfusion injury [61], and (ii) fetuin-A which is expressed in the cytoplasm of renal tubular cells, especially those detached from the basal lamina [62]. In the setting of decompensated cirrhosis, urine exosome protein characterization in AKI patients revealed increased secretion of maltase glucoamylase, a renal brush border disaccharidase [63]. In renal transplant patients, Sigdel et al. observed that acute rejection and BK-virus-associated nephropathy, two main causes of acute loss of renal function in this population, present with different urinary exosomal protein expression profiles; higher abundance of CLCA1, PROS1, KIAA0753, and ApoM was linked to acute rejection [64].

### 3.5. Nephrotic Syndrome

Nephrotic syndrome represents a constellation of symptoms including peripheral edema, heavy proteinuria, hypoalbuminemia, and often hyperlipidemia and is thought to result from increased glomerular permeability to albumin and other plasma proteins [65]. The various causes of nephrotic syndrome can be grouped together according to the microscopic pattern of injury into the following: minimal change disease (MCD), focal segmental glomerulosclerosis (FGSG), membranous glomerulonephritis, mesangiocapillary glomerulonephritis, and other, such as amyloidosis [65].

In the pediatric population, a urinary exosomal miRNA profile of upregulated miR-194-5p, miR-146b-5p, miR-378a-3p, miR-23b-3p, and miR-30a-5p has been identified in various histological patterns of injury in idiopathic nephrotic syndrome [66]. At the same time, miR-193a levels may be useful in distinguishing between pediatric primary FGSG and MCD [67]. In addition, the detection of WT-1, a marker of podocytal injury, may aid in diagnosing FGSG when also steroid-sensitive nephrotic syndrome is considered [68]. WT-1 mRNA is generally not detected in the urine of MCD patients, but it is isolated in cases of diabetic nephropathy. Other presumed markers of podocytal injury (podocin, Actn4, CD2AP and Nephrin mRNA) seem insufficient to help differentiate between those two conditions [49].

Crescent formation, the hallmark of RPGN, has been associated with the presence of fibroblast-specific protein 1 (FSP1), a cytosolic protein expressed by increased number of renal cells in kidneys exhibiting ongoing injury [69]. In idiopathic membranous nephropathy, upregulation of blood and urinary MUC3A circular RNA (circRNA) and various small nucleolar RNAs (snoRNAs) such as SNORA51, SNORA31, SNORA70, SNORA75, and SNORD112 has been reported [70]. Lastly, Ramirez-Alvarado et al. demonstrated the presence of amyloidogenic light chains in urinary exosomes of patients with amyloidosis but not in patients with multiple myeloma without amyloidosis [71, 72].

### 3.6. Nephritic Syndrome

Nephritic syndrome, defined by the presence of hematuria in association with hypertension, oliguria, fluid retention, and a decline in renal function, is an inflammatory process with a histological picture of glomerular basement membrane ruptures and usually diagnosed by renal biopsy. Common causes of nephritic syndrome include anti-GMB disease, IgA nephropathy, and lupus nephritis.

In IgA nephropathy, increased expression of urinary exosomal miR-215-5p and miR-378i and decreased expression of miR-29c and miR-205-5p have been described compared to healthy individuals [73]. Higher levels of aminopeptidase N, vasorin precursor, *α*-1-antitrypsin, and ceruloplasmin have been used to distinguish between IgA and thin basement membrane nephropathy which is another common cause of glomerular hematuria [74]. A decrease in urinary exosomal miR-29c may indicate podocytal injury [75], whereas an increase in CCL2 mRNA may represent tubulointerstitial inflammation and C3 deposition [76].

Lupus nephritis is characterized by downregulation of urinary exosomal miR-29c [77] and upregulation of miR-146a [78], miR-150, and miR-21 [77]. A decrease in miR-21 along with let-7a miRNA precursor may indicate disease flare [79]; an increase in urinary exosomal miR-3135b, miR-654-5p, and miR-146a-5p has been described in cellular crescent formation of lupus nephritis [80]. Conversely, higher urinary exosomal levels of miR-31, miR-107, and miR-135b-5p are associated with a better response to treatment [81]. Lower levels of miR-29c have been correlated with both renal fibrosis, even without a decline in renal function [82], and podocyte injury [75].

### 3.7. Genetic Disorders

Cystic kidney diseases (CKD) are heterogeneous in origin, distribution, and pathogenesis; many are related to genetic defects. Autosomal dominant polycystic kidney disease (ADPKD), the most common inherited CKD, mainly results from PKD1 mutations. Hogan et al. described lower PKD1 and higher transmembrane protein 2 (TMEM2) urinary exosomal protein secretion in ADPKD, suggesting that the PKD1/TMEM2 ratio may have some diagnostic utility [83]. Thirty differentially expressed urinary exosomal proteins between ADPKD patients and healthy controls have been identified: urinary periplakin, envoplakin, villin-1, and complement C3 and C9 were more abundant in ADPKD [84]. Additionally, lower AQP-2 and higher APO-A1 levels were correlated with eGFR decline [85]. There is evidence that urinary exosomes in ADPKD individuals may have a different surface glycosylation profile than that of healthy individuals [86].

Exosome isolation and characterization have assisted in the diagnostic challenge to differentiate between medullary sponge kidney (MSK), a cause of medullary nephrocalcinosis, and idiopathic calcium nephrolithiasis. Bruschi et al. found that higher FCN1 and C4PBP, as well as lower MASP2 serum exosome protein levels, were positively associated with MSK [87]. Furthermore, a lower urinary CD133 level seems to favor the diagnosis of MSK over ADPKD [88]. Nephronophthisis, another renal medullary cystic disorder, also presents with a distinct urinary exosomal protein profile [89].

A pilot study with patients with cystinuria highlighted that 165 urinary exosomal proteins, analyzed by mass spectrometry, could be utilized to identify patients and also determine the severity of disease [90]. In two patients with Bartter syndrome type 1, Gonzales et al. noted the absence from urinary exosomes of NKCC2, the protein encoded by the SLC12A1 gene which mutated in this disease [91].

## 4. Discussion

Treatment optimization of renal disease depends on the availability of diagnostic and prognostic biomarkers. The use of renal biopsy, which remains the gold standard in the diagnosis of kidney disease, is also affected by potentially serious postoperative complications and the possibility of improper or nonrepresentative sampling.

### 4.1. Advantages of Exosomal Biomarkers in Renal Disease

It is evident that exosomes may be the solution to finding accurate renal disease biomarkers without the need for invasive procedures. Proteomic profiling of urinary exosomes by mass spectroscopy and subsequent computational analysis, reveals an abundance of proteins implicated in pathophysiologic processes such as sodium ion transport, immune activation, and epithelial cell differentiation [92]. Additionally, a significant portion of exosomal protein cargo plays a well-established role in glomerular physiology. Some examples of relevant proteins isolated in exosomes include podocalyxin, lysosomal-associated membrane protein 2, Src substrate cortactin, Rab 23, ENPP6, ezrin, complement C4B, agrin, FAT4, CD59, talin 1, syntenin 1, neprilysin, Na^+^/H^+^ exchange regulatory cofactor 2, and angiotensin-converting enzyme [93]. Production of pathologic proteins regulated by defective genes in exosomes from certain genetic renal diseases may be either decreased (PKD1 in ADPKD) [83] or totally absent (SLC12A1 in Bartter syndrome type 1) [91].

**Figure 1:**
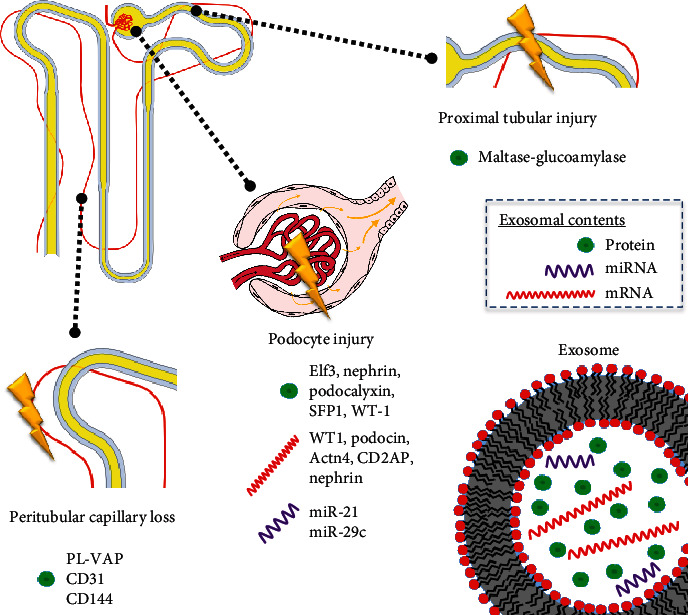
Exosomal biomarkers reflecting alterations in compartments of the nephron.

Another advantage of exosomes as potential biomarkers is the expression of markers that are specific for their cell of origin that allows the tracking of alterations in specific cellular compartments within a tissue (Figure 1). Hogan et al. identified in urine exosomal cargo molecules specific for their place of biogenesis which includes mesangial and subendothelial cells, proximal tubule cells, glomerular basement membrane (GBM), podocytes and slit diaphragm, podocyte-GBM interface, glomerular endothelial cells, and capillary loops [93]. It is of great interest that, for example, an increase in podocyte- or endothelial-derived exosomes which were determined by the presence of podocytal proteins podocin [49], nephrin, and podocalyxin [43, 60] may indicate podocytal damage. Similarly, higher levels of exosomal endothelial proteins PL-VAP, CD31, and CD144 [59] suggest endothelial damage. Interestingly, urinary exosomal miR-200b was associated with renal fibrosis only when measured in CD13+ (i.e., nonproximal renal tubule-derived) exosomes in CKD [45]; this indicates that a biomarker may be of clinical significance when it is associated with exosomes derived from a specific cell population.

### 4.2. Challenges in the Use of Exosomal Biomarkers in Renal Disease

Despite all the theoretical advantages of exosomal biomarkers, there are many challenges, both technical and translational that need to be addressed before the routine application in the clinical practice.

#### 4.2.1. Technical Challenges

The main challenge in exosome isolation is to differentiate exosomes from other EVs. Unfortunately, to date, there is no single isolation technique that guarantees purity, speed, cost-effectiveness, and ability to process large sample volumes at the same time. Ultracentrifugation-based techniques are inexpensive with a low contamination risk and are suitable for large volume preparation; however, they require expensive nonportable equipment and are labor-consuming, making them unsuitable for small volume sampling. Additionally, high centrifugation speeds may mechanically damage EVs [15]. Ultrafiltration is a low-cost, fast and portable procedure, but the end result suffers from moderate purity when used alone. The shear forces that develop during this process can lead to potential loss of exosomes due to entrapment in the filtration membrane [15]. Tangential flow filtration is a promising filtration technique that avoids membrane clogging and mechanical EV damage, while allowing for processing of large volumes in a time-efficient manner [16]. Size-exclusion chromatography is a quick, reproducible method, suitable for both large and small sample volumes resulting in highly pure EVs, but it is limited by the relatively high cost and the necessity for an additional exosome enrichment method [15]. Polymer precipitation is an easy-to-use technique, also versatile for both large and small sample volumes; nonetheless, it requires extended processing times, and exosomal concentrates may be contaminated with protein aggregates, other extracellular vesicles and polymeric contaminants [15]. Immunoaffinity capture is an easy-to-use, high-purity method, able to separate exosomes based on their origin, which may be appealing in the case of urinary exosomal biomarkers; however, the required antibodies may be costly, and the technique is dependable on the specificity of the exosomal marker which is used for exosome identification and cannot be used in larger sample volumes. This method also requires an extra step for exosome elution, which may damage the exosomal structure [15]. Microfluidics-based techniques are highly efficient, cost-effective, portable, and easily automated but suffer from limited sample capacity [15]. Any new exosome isolation technology should be validated before it becomes available for clinical use, a process which is oftentimes lengthy. Although there are reproducibility concerns due to the variability of isolation methods reported in the literature, the development of an exosome-specific nomenclature with descriptive definitions has been an important step towards improved standardization of results among studies [94].

#### 4.2.2. Biological and Clinical Challenges

The exosomal cargo is speculated to be reflective of complex intracellular changes. However, it is unclear and dependent on the condition whether exosomal biomarkers can be more sensitive for the detection of a pathologic process than nonexosomal biomarkers. In general, exosomal and nonexosomal EV cargoes can overlap considerably but are not identical. For example, analysis of the proteomic composition of urinary EVs has revealed that some proteins are detected exclusively either in microvesicles or exosomes [87]. In lupus nephritis and more specifically in active disease, larger quantities of miRNA biomarkers were identified in urinary exosomes than in the cell-free fraction of urine preceding exosome isolation [78]. Some biomarkers seem to better correlate with the clinical condition or variable in question when measured in the exosomal content; for instance, exosomal ceruloplasmin and/or gelatinase are superior in reflecting changes in renal tissue compared to their direct urine measurements [38, 95]. However, other biomarkers such as the urinary NGAL and IL18 proteins in patients after renal transplantation correlated to day seven post op creatinine reduction ratio, whereas the corresponding urinary exosomal transcripts did not [96].

Another consideration is which biofluid is optimal for exosomal biomarkers in renal disease diagnostics. The vast majority of biomarkers examined in this review were identified in urinary exosomes and very few in serum. Other biofluids such as peritoneal dialysis aspirate contain exosomes which could carry biomarkers associated with membrane failure [97]. It has been observed that urinary and blood exosomal contents are very different [98]. There are currently very few studies that compare the usability of urinary vs. nonurinary exosomes in renal disease. However, Sun et al. noted that urinary endothelial-derived exosomes identified renal microcirculation injury better than systemically circulating endothelial-derived exosomes in hypertensive patients [59].

The diurnal variations of urine consistency should also be accounted for in the evaluation of urinary biomarkers. Urine creatinine is commonly used to normalize the values of soluble urinary biomarkers, but its relevance to exosomal biomarkers remains unknown [99]. As far as timing of biosampling is concerned, a circadian pattern in urinary exosomal excretion has been observed in rats with peak concentrations occurring between 19:00 and 23:00 hours, although the circadian variation seems to be normalized with TSG101 protein levels [100]. All types of circulating EVs are reduced following dialysis [101]. Fernández-LLama et al. suggest that Tamm-Horsfall protein levels can be useful in the normalization of urinary exosome concentration in spot urine samples [102].

Lastly, comorbidities should also be taken into account. Changes in circulating and urinary exosomal contents have been reported in patients treated with antihypertensive agents [103] or cyclosporine [104], respectively. It is worth mentioning though that even serious proteinuria secondary to glomerular damage does not seem to affect the concentration of urinary exosomes [105].

## 5. Conclusion

Exosomes represent a valuable source of candidate diagnostic and/or prognostic biomarkers for a variety of renal conditions. Their potential to reflect changes in specific cellular compartments of the nephron is of particular interest. Exosomes, particularly urinary ones, may provide a dynamic image of the processes taking place in the affected renal tissue. Exosomal biomarkers unlike renal biopsies are not limited by the possibility of obtaining unrepresentative sampling. Exosomal purification and analysis require minimally to noninvasive techniques depending on the biofluid of interest, and exosomal isolation technology is constantly improving. This allows for serial analyses in follow-up clinical visits for comparison. Moreover, exosomal miRNAs have a potential diagnostic and therapeutic potential mainly to their active role in disease pathophysiology. In many cases, exosomal biomarkers may complement renal biopsy in risk stratification and prognostic evaluation. Clinical correlations of currently available data on exosomes in kidney disease are difficult to make currently because of a considerably low to moderate sample size in most research efforts found in the literature and the lack of a standardized methodology of exosome isolation that prevents the direct comparison of study results. Further work is warranted in order to identify accurate and reliable exosomal biomarkers that could complement or replace currently available diagnostic tools. Although great progress has been achieved in exosome research so far, further work is warranted in order to identify accurate and reliable exosomal biomarkers that could complement or replace currently available diagnostic tools.

## Figures and Tables

**Table 1:** Urinary exosomal biomarkers potentially useful in the recognition of early damage patients (pts)/control (ctr).

Condition	Potential exosomal biomarker	Study subjects	Reference
CKD	Ceruloplasmin ↑	51 pts-15 ctrs; rats	[38]
	miR-181a-5p ↑, among 30 differentially expressed ncRNAs	15 pts-10 ctrs	[39]
	Exosomal miR-451 ↑	38 pts-23 ctrs	[40]

Lupus nephritis	miR-150 and miR-21 ↑; miR-29c ↓	45 pts-20 ctrs	[77]
	miR-146a ↑	38 pts-12 ctrs	[78]

Diabetic kidney disease	Among 22 proteins: MASP2 and CALB1 ↑; S100A8 and S100A9 ↓	60 pts-15 ctrs	[53]
	miR-21-5p ↑; miR-30b-5p ↓	66 pts	[51]
	miR-15b, miR-34a, and miR-636	54 pts-12 ctrs	[52]
	Myeloblastin, elafin, cystatin B and neutrophil gelatinase-associated Lipocalin ↑	37 pts-12 ctrs	[54]
	Regucalcin ↓	4 pts-3 ctrs; rats	[55]

Nephronophthisis	156 differentially expressed proteins	12 pts-12 ctrs	[89]

Acute kidney injury in critical disease	Activating transcriptional factor 3 ↑	8 pts-8 ctrs; mice	[61]
	Fetuin-A ↑	6 pts; rats	[62]

**Table 2:** Urinary exosomal biomarkers potentially useful in disease monitoring and/or management patients (pts)/control (ctr).

Condition		Potential exosomal biomarker	Study subjects	Reference
Lupus nephritis	Prediction of clinical response	miR-31, miR-107, and miR-135b-5p ↑	57 pts	[81]
	Disease flare	let-7a and miR-21 ↓	34 pts	[79]
	Cellular crescent formation in type IV lupus nephritis	miR-3135b, miR-654-5p, and miR-146a-5p ↑	14 pts-3 ctrs	[80]

IgA nephropathy	Tubulointerstitial inflammation and C3 deposition	CCL2 mRNA ↑	55 pts-24 ctrs	[76]

Nephropathy in type 1 diabetes	Various degrees of albuminuria	Various differentially expressed miRNAs	48 pts	[56]

Nephropathy in type 2 diabetes	Decline in renal function	Uromodulin mRNA ↑	242 pts and ctrs	[57]
	Progression of albuminuria	C-megalin	33 pts-11 ctrs	[58]
	Macroalbuminuria	miR-362-3p, miR-877-3p, and miR-150-5p ↑; urinary miR-15a-5p ↓	5 pts-5 ctrs	[106]

AL amyloidosis	Active amyloid formation	Light chain oligomers	4 pts-1 ctr	[71]
			13 pts-1 ctr	[72]

Autosomal dominant polycystic kidney disease	eGFR decline	AQP-2 ↓; APO-A1 ↑	46 pts-11 ctrs	[85]

Cystinuria	eGFR value	165 differentially expressed proteins	8 pts-10 ctrs	[90]

**Table 3:** Exosomal biomarkers associated with specific etiological factors of renal disease patients (pts)/control (ctr).

Condition		Potential exosomal biomarker	Study subjects	Reference
Medullary sponge kidney	vs. idiopathic calcium nephrolithiasis	Blood FCN1 and C4BPB proteins ↑; blood MASP2 protein ↓	15 pts-15 ctrs	[87]
	vs. autosomal dominant polycystic kidney disease	Mainly urinary CD133 ↓, among 34 discriminative urinary EV proteins	15 pts-15 ctrs	[88]

Autosomal dominant polycystic kidney disease		Urinary periplakin, envoplakin, villin-1, and complement C3 and C9 ↑, among 30 proteins	34 pts-32 ctrs	[84]
		Urinary PC1/TMEM2 or PC2/TMEM2 ↓	13 pts-18 ctrs	[83]

Diabetic nephropathy	vs. minimal change nephrotic syndrome	Urinary WT1 mRNA ↑	20 pts-5 ctrs	[49]

Cadmium-induced nephrotoxicity		Blood MT1DP lncRNA ↑	100 persons	[107]

Idiopathic membranous nephropathy		Blood and urinary MUC3A circRNA and various snoRNAs ↑	10 pts-10 ctrs	[70]

Pediatric idiopathic nephrotic syndrome		Urinary miR-194-5p, miR-146b-5p, miR-378a-3p, miR-23b-3p, and miR-30a-5p ↑	129 pts-126 ctrs	[66]

Pediatric primary focal segmental glomerulosclerosis	vs. minimal change disease	Urinary miR-193a	13 pts	[67]

IgA nephropathy	vs. thin basement membrane nephropathy	Urinary miR-215-5p and miR-378i ↑; urinary miR-29c and miR-205-5p ↓	18 pts-18 ctrs	[73]
		Urinary aminopeptidase N, vasorin precursor, *α*-1-antitrypsin, and ceruloplasmin ↑	12 pts-7 ctrs	[74]

Acute rejection	vs. BK nephropathy or chronic allograft injury	Urinary CLCA1, PROS1, KIAA0753, and ApoM ↑	30 pts-20 ctrs	[64]

Focal segmental glomerulosclerosis	vs. steroid-sensitive nephrotic syndrome	Urinary WT-1 ↑	25 pts-5 ctrs	[68]

Bartter syndrome type 1		Urinary NKCC2 protein ↓	2 pts	[91]

**Table 4:** Urinary exosomal biomarkers associated with injury localized to a specific cellular or subcellular component of the nephron patients (pts)/control (ctr).

Condition		Potential exosomal biomarker	Study subjects	Reference
Podocyte injury	In diabetic nephropathy	Elf3 protein ↑	50 pts-5 ctrs	[48]
		WT1, podocin, Actn4, CD2AP, and nephrin mRNA ↑	20 pts-5 ctrs	[49]
	In minimal change nephrotic syndrome	Podocin, Actn4, CD2AP, and nephrin mRNA ↑		
	In metabolic syndrome-related kidney disease	Podocyte-derived exosomes (nephrin+/podocalyxin+) ↑	16 pts-15 ctrs	[43]
	In CKD	miR-21 ↑	41 pts-5 ctrs	[44]
	In cellular crescent formation	SFP1 ↑	37 pts	[69]
	In renovascular hypertension	Podocyte-derived exosomes (nephrin+/podocalyxin+) ↑	31 pts-45 ctrs	[60]
	In lupus nephritis	miR-29c ↓	24 pts-8 ctrs; mice	[75]
	In IgA nephropathy			
	In focal segmental glomerulosclerosis	WT-1 ↑	25 pts-5 ctrs	[68]

Proximal tubular injury	In decompensated cirrhosis	Maltase glucoamylase ↑	24 pts	[63]

Renal fibrosis	In CKD	Nonproximal tubule-derived miR-200b ↑	38 pts	[45]
		miR-29c ↓	32 pts-7 and ctrs	[46]
		CD2AP mRNA ↓	32 pts-7 ctrs	[47]
	In lupus nephritis	miR-29c ↓	47pts-20 ctrs	[82]

Peritubular capillary loss	In hypertension	Endothelial-derived EVs (PL-VAP+/CD31+/CD144+) ↑	38 pts-14 ctrs	[59]

Mitochondrial dysfunction	In diabetic nephropathy	12 mitochondria-specific metabolites ↓	149 pts-23 ctrs	[50]
